# Supplementary material for: The elimination of trachoma as a public health problem in Togo: Successes and challenges
Source: PLoS Negl Trop Dis. 2023 Jul 18;17(7):e0011444. doi: 10.1371/journal.pntd.0011444 (PMC10353810; doi:10.1371/journal.pntd.0011444)
Supplement: Attachment — Submitted filename: Authors responses.docx [file pntd.0011444.s001.docx]

1- **Title**

**Editorial comments**

Title of paper requires revision since this manuscript focuses on trachoma. You might want to review the title to be more generic on the 4 eliminated NTDs but specific on trachoma, for example: “Togo’s success story in eliminating four NTDs as a public health problem within a decade: Lessons learnt from trachoma elimination"

**Authors**: Thank you for this proposition. We have revised the title to be specific to trachoma.

Title: The elimination of trachoma as a public health problem in Togo: Successes and challenges

I note that the authors are primarily French speakers thus some context has possibly been lost in the English translations.  As highlighted by the reviewers, I suggest that the manuscript be copy edited to correct the language and also include the appropriate English acronyms.

**Authors**: Thank you for this suggestion. The manuscript will be revised to correct language and include appropriate English acronyms.

2- **Methods**

**Reviewer #1** This article is relevant and could be a good reference for countries that are still struggling to achieve trachoma elimination as a public health problem.  However, the paper is not very well written and lacks coherence and smooth flow of the historical development of the program.  From the outset, the title of the paper doesn't appear to be appropriate.  The article is all about trachoma elimination in Togo but not clear why it's titled as such.  The authors can discuss the success stories of other programs in the introduction or in the Discussion sections but the title of the paper should only be focused on trachoma elimination in Togo.

**Authors**: Thank you for this proposition. We have revised the title to be specific to trachoma.

Title: The elimination of trachoma as a public health problem in Togo: Successes and challenges

**Reviewer #1** One other general comment is that the paper has to be rewritten by a native English language speaker.  There are some accronyms that I suspect have come from a French language.  Examples are; CT, PNMTN, MSHPAUS, PNLC, MTD, TDM, FT, ASC, CHANCE, OSI, TSO, ICMR, IRMS, NCDFPs, OSI, etc. These should be replaced by the appropriate English accronyms

**Authors**: Thank you for this suggestion. The manuscript will be revised to correct language and include appropriate English acronyms.

**Reviewer #1** The baseline trachoma prevalence data were not appropriately presented.  On page 7, it says the prevalence of trachoma in the Binah district, Kara region was 60 - 80%.  Looking at the following results of the survey in 2009, which was on average less than 10%, the prevalence reported for Binah district doesn't seem accurate.  There must have been a methodological error introduced by the Integrated Threshold Mapping approach.  There is no detailed summary table provided for the results of the 2006 survey.  That should be corrected.

Authors: Thank you for this comment.

There was no methodological error introduced by the integrated threshold mapping approach. Because in 2009, a survey was conducted in both school and community facilities in 14 of the 15 health districts in the three northern regions of Togo (Centrale, Kara and Savanes) with the exception of the Binah district (where a similar survey had already been conducted in 2006). Therefore, there is no prevalence of trachoma reported for Binah District in 2009 (Table 1)

**Reviewer #1** There are lots of omissions or wrongly coined or incomplete statements.  For example, in many places, it says "WHO awarded Togo for becoming ...." Awarded what? 

**Authors:** Thank you for this comment. it's not an award, it's a distinction. We will correct this in the revised version

**Reviewer #1** In relation to TT management, the phrase "eyelash removal" has been repeatedly used.  This has to be replaced by the correct term "epilation".

**Authors**: We thank the reviewer for these corrections. We will correct this in the revised version

**Reviewer #1** On Table 3, the heading has to change; "Results of TFs and TTs" is not appropriate use of the terms
"CHANCE" should be replaced by "SAFE" strategy.

**Authors**: We thank the reviewer for these corrections. We will correct this in the revised version

**Reviewer #1** On page 15, An independent "case review panel" should be replaced by "Dossier review panel".

**Authors**: We thank the reviewer for this correction. We will correct this in the revised version

"An independent regional dossier review panel convened by WHO reviewed the case and confirmed" that the criteria for elimination were met

**Reviewer #2:** The objectives of the study are clearly stipulated, and the article follows standard trachoma methodology guidelines.
No issues

**Authors:** Thank you for this comment.

**Reviewer #3**: This article is based mainly on events that took place under the trachoma elimination program in Togo so even though it has clear objectives, no hypothesis is stated and cannot be assessed against a study design, sample sizes and methods and did not involve any statistical analysis. it is just a review of processes leading to the attainment of trachoma elimination together with 3 other diseases within 11 years.

**Authors:** Thank you for this comment. Indeed, it is just a review of processes leading to the attainment of trachoma elimination. We believe that this can be useful for other countries in the process of elimination.

3- **Results**

**Reviewer #1**: I have discussed some of the Results section already in the above section.  It requires rewriting.

**Authors**: Thank you for this comment. Indeed, it was about the prevalence reported in the district of Binah in 2009 which would not be exact. However, in 2009, a survey was conducted in both school and community facilities in 14 of the 15 health districts in the three northern regions of Togo (Centrale, Kara and Savanes) with the exception of the Binah district (where a similar survey had already been conducted in 2006). Therefore, there is no prevalence of trachoma reported for Binah District in 2009 (Table 1)

**Reviewer #2**: results are descriptive and clear

**Authors:** Thank you for this comment.

**Reviewer #3**: The article involved the presentation of already analysed survey data and presentation in a very simple form to indicate how decisions toward elimination and certification were taken. In my opinion the content of the paper does not adequate justify the topic and so the topic or the content needs to be reviewed to fall in alignment. The content only focuses on trachoma elimination with little mention of the other three diseases.

**Authors**: Thank you for this comment. We have revised the title to be focused to trachoma

Title: The elimination of trachoma as a public health problem in Togo: Successes and challenges

4- **Conclusion**

**Reviewer #1**: The conclusion is ok but but needs rewriting.  There shouldn't be quoted statements in the conclusion.

**Authors**: Thank you for this comment. We have removed the quotes in the conclusion

**Reviewer #2:** conclusion is well supported by findings. The article has public health relevance for neglected tropical diseases

**Reviewer #3:** The conclusions are supported by the presented data, however the limitations of the process and events towards these achievements are not adequately highlighted in the article.

**Authors:** Thank you for this comment.

**Authors**: Thank you for this comment.

5- **Editorial and Data Presentation Modifications**

Use this section for editorial suggestions as well as relatively minor modifications of existing data that would enhance clarity. If the only modifications needed are minor and/or editorial, you may wish to recommend “Minor Revision” or “Accept”.

**Reviewer #1**: Major revision required.

**Authors:** Thank you for this comment.

**Reviewer #2**: Minor comments:
The article has several typos that need to be corrected.
Author summary
Line 47. Type which was to be which was
Line 49 type screen and
Line 50 should be achieved in all
Table 1 has a larger different font and format. please revise 
Line 186 -191; can SAFE be used instead of CHANCE as the article in is English and not French?

**Authors:** We thank the reviewer for these propositions. These corrections will be integrated in the revised version.

**Reviewer #3**: On page 10, line 116, the heading “Detection of suspected TT cases during CT scans from 2015 to 2017” needs to be reviewed and clarified since CT in the manuscript apparently stands for community treatment and not computer tomography as it appears here. There are a few other editorial errors that require attention.

**Authors:** We thank the reviewer for these propositions. These corrections will be integrated in the revised version.

6- **Summary and General Comments**

Use this section to provide overall comments, discuss strengths/weaknesses of the study, novelty, significance, general execution and scholarship. You may also include additional comments for the author, including concerns about dual publication, research ethics, or publication ethics. If requesting major revision, please articulate the new experiments that are needed.

**Reviewer #1:** The article doesn't merit publication in its current form.

**Authors:** Thank you for this comment. The corrections will be integrated in the revised version.

**Reviewer #2**:

This is a god article and will be useful for other countries nearing elimination. it should be noted that additional countries like Malawi have also declared Trachoma elimination  in Sept 2022, so it may need updating the figures

**Authors:** We thank the reviewer for this information. It will be included in the revised version.

Minor comments:
The article has several typos that need to be corrected.
Author summary
Line 47. Type whichwas to be which was
Line 49 type screenand
Line 50 should be achieved in all
Table 1has a larger different font and format . please revise 
Line 186 -191; can SAFE be used instead of CHANCE as the article in is English and not French?

**Authors:** We thank the reviewer for these propositions. These corrections will be integrated in the revised version.

**Reviewer #3:**

Review Comments: How did Togo, a West African country, eliminate lymphatic filariasis, dracunculiasis, human African trypanosomiasis, and trachoma as a public health problem in only 11 years? Sharing experience: The case of trachoma. This is a great paper about an achievement or feat worth celebrating in global health. Even though a country’s achievements, various partners collaboratively contributed technically and financially to make this a reality with the government being the centerpiece in driving these efforts with insightful lessons for other disease programs in Togo and other sub-Saharan African countries. The paper acknowledges the different roles played by the different organizations and civil society with the WHO being a significant partner in the scheme of affairs. The role of government in the scheme of affairs does not come out clearly and strongly to me.There have been several technical opportunities, in this journey, together with challenges, and learning to drive other programs within Togo and for other countries in sub-Saharan Africa and elsewhere globally. In my reading of the manuscript, I realized there were no surveys to validate the data already collected during surveys conducted in 2006, 2009, and 2011.
Mapping data was also not included in the write-up and how it was used in planning treatment to enable follow-up surveys and demonstration of the trajectory of transmission over the period. It is however clear that adequate baseline data was collected to guide the program. Various methods were adapted for the surveys undertaken in 2006, 2009, 2011, and 2016. The guidelines applied and some analysis of the survey methodologies to bring out differences that could have impacted the results obtained and recommended methods moving forward would have been insightful and informative. Readers would be interested to know what happened between the period from 1989, with the establishment of the NCCP till 2016 when the mapping or baseline surveys were conducted which is also unclear in the manuscript.

**Authors:** Thank you for this comment.

On page 10, line 116, the heading “Detection of suspected TT cases during CT scans from 2015 to 2017” needs to be reviewed and clarified since CT in the manuscript apparently stands for community treatment and not computer tomography as it appears here.

There are a few other editorial errors that require attention.

In all this is an interesting and insightful paper on an important and critical subject in the history of the Global NTD Program with great lessons to inform many public health programs in sub-Saharan Africa. I recommend this article for publication after addressing the comments from the review.
Thank you for the opportunity to review this manuscript.

**Authors:** We thank the reviewer for these propositions. These corrections will be integrated in the revised version.

**Authors:** Thank you for this comment. We thank you for your acceptance to review this manuscript.
