# Supplementary material for: The elimination of trachoma as a public health problem in Togo: Successes and challenges
Source: PLoS Negl Trop Dis. 2023 Jul 18;17(7):e0011444. doi: 10.1371/journal.pntd.0011444 (PMC10353810; doi:10.1371/journal.pntd.0011444)
Supplement: Attachment — Submitted filename: Authors responses.docx [file pntd.0011444.s002.docx]

**Responses to all review comments and description of the changes we have made in the manuscript**

1- **Methods**

**Reviewer #2:** (No Response)

**Reviewer #3:** The objectives are clearly stated but because it's a review paper there is no stated hypothesis. The endemic populations are well described together with the studies or operational research activities that were undertaken there.

**Authors:** Thank you for this comment

2- **Results**

**Reviewer #2:** (No Response)

**Reviewer #3**: The analysis of the data which is mainly retrospective has been well done and presented. The data employed for the drafting of the manuscript was basically longitudinal retrospective programmatic review data already analyzed and applied for programmatic decision making which is in order. This data has been presented in a logical chronological manner which should make a lot of sense to any reader. The tables used are of fair quality and provide a lot of clarity to the data analyzed and presented.

**Authors:** Thank you for this comment.

3- **Conclusion**

**Reviewer #2**: (No Response)

**Reviewer #3:** Being a review paper, the authors are limited in identifying limitations but the main limitation as I see it is the use of retrospective programme data the authors have limited control over at the time of writing up.

**Authors:** Thank you for this very pertinent observation. Indeed, the main limitation is the use of retrospective data over which we had limited control at the time of writing.

This difficulty must be noted. We will mention it in the revised version.

4- **Editorial and Data Presentation Modifications**

**Reviewer #2**: (No Response)

**Reviewer #3**: The revised version of the paper as it stands now is a marked improvement of the original version. It is well-written, flows well and requires veery little editorial work.

**Authors:** Thank you for this comment.

5- **Summary and General Comments**

**Reviewer #2: Review**

Overall most concerns have been addressed. However the paper still has a lot of typos and need a copy editor.

**Abstract:**

Line 22 ADD dossier to state Country’s dossier submission

**Authors:** Thank you for the correction. This correction will be incorporated in the revised version.

A review and compilation of data and information contained in the country's dossier for submission to World Health Organization for validation of trachoma elimination as a public health problem was conducted

Line 25. Separate alsoincluded to also included

**Authors:** Thank you for the correction. We will separate alsoincluded to also included.

Data from national and local surveillance systems and reports on actions taken after achieving the elimination target were **also included**.

Line 27 . remove has.

All results should be stated in the past tense. Line 28 should read was < than 5% and line 30 was less than 0.2%

**Authors:** Thank you for the correction. This correction will be incorporated in the revised version.

The prevalence of follicular trachoma among children aged 1-9 years **was** <5% in all nationally defined administrative units suspected of having trachoma after stopping mass treatment for at least 2 years. The prevalence of trichiasis among persons aged 15 years and older **was** less than 0.2% in all administrative units previously endemic for trachoma and evidence of the ability to manage incident cases of emerging trichiasis in the community has been demonstrated

Sentence 37 starting with BUT . please rephrase

**Author Summary**

Line 47 . screen and manage . there is typo

**Authors:** Thank you for the correction. We will separate scree nand maage to screen and manage.

Between 2006 and 2016, different surveys were conducted to identify trachoma endemic areas, **screen and manage** Trachomatous trichiasis cases.

**Introduction**

Line 71 typo whichwas

**Authors:** Thank you for the correction. We will separate whichwas to which was.

During the 72^nd^ Session of the WHO Regional Committee for Africa held in Lomé, Togo, from 22 to 26 August 2022, WHO honored Togo for becoming the first country in the world to eliminate four neglected tropical diseases in only 11 years namely dracunculiasis (Guinea worm), lymphatic filariasis, human African trypanosomiasis (sleeping sickness) and trachoma, the last of **which was** eliminated in May 2022

Line 72 five to be replaced by fifth. Line 73 Malawi was declared in September after Togo. please change

**Authors:** Thank you for the correction. We checked. Indeed, Togo was certified (in May 2022) before Malawi (in September 2022).

These corrections will be integrated in the revised version.

**Methods**

line 86. We did not require consent. if this true? Even if this was secondary data, consent /permission from Ministry of Health to use it was needed . please clarify that you did not obtain consent

**Authors:** Thank you for this comment. This correction will be incorporated in the revised version.

This study used anonymized case data, which were collected by the National Neglected Tropical Disease Program as part of its program surveillance activities. In this study, no interventions were performed (neither diagnostic, nor therapeutic, nor survey). We relied solely on a retrospective collection of anonymous cases authorized by the National Neglected Tropical Disease Program. We have not been in contact with any individual. We do not require consent and could not obtain it in this context.

Line 102. give a reference

**Authors:** Thank you for this proposition. We will add a reference in the revised version

Dorkenoo AM, Bronzan RN, Ayena KD, Anthony G, Agbo YM, Sognikin KS, Dogbe KS, Amza A, Sodahlon Y, Mathieu E. Nationwide integrated mapping of three neglected tropical diseases in Togo: countrywide implementation of a novel approach. Trop Med Int Health. 2012 Jul;17(7):896-903. doi: 10.1111/j.1365-3156.2012.03004.x

Line 113 -	was this the WHO standardized recognized methodology? Please clarify the methodology. Did the survey also address the issue of TT ? this is not mentioned?

**Authors:** Thank you for this comment.

This is not the standardized methodology recognized by the WHO. It was a community-based cluster survey conducted in the three districts (Blitta, Sotouboua, and Binah) where TF appeared to be prevalent at levels above the elimination threshold in the 2006 and 2009 surveys.

The 2011 survey searched for TF cases among children aged 1-9 years and TT among women aged 15 years and older in these three districts.

We will clarify it in the revised version

Line 119 : typo resultsof

**Authors:** Thank you for the correction. We will separate resultsof to results of.

These two regions were chosen based on the **results of** the 2009 and 2011 surveys

Line 132 : collated ? please explain ?

**Authors:** Thank you for this comment.

Survey results from 2006, 2009, and 2011, mass treatment data, routine health facility data from 2014 to 2016, and TT case data from the project from June 2015 to December 2016 were **compiled**. Taking all these data into account, 07 health districts from three regions were selected to Tropical Data Survey.

Line 133 : typo -allthese

**Authors:** Thank you for the correction. This correction will be incorporated in the revised version.

Taking **all these** data into account, 07 health districts from three regions were selected.

It is not clear when the surveys with Tropical data were conducted. is it after 2016 ? please clarify ?

**Authors:** Thank you for this comment.

Survey with Tropical data were conducted after 2016, in 2017.

We will clarify it in the revised version

Line 172 is not clear. What is 159 eyes to cure ?

**Authors:** Thank you for the correction.

The project diagnosed 108 people with TT. 159 eyes were affected. 116 eyes received surgery while 43 eyes received epilation of eyelashes for those who refused surgery.

This correction will be incorporated in the revised version.

Line 179 were surgered ? not correct English

**Authors:** Thank you for the correction.

19 of them were **operated** while 6 refused surgery and therefore benefited from epilation of eyelashes.

This correction will be incorporated in the revised version.

Line 227 againstonchocerciasis – typo

**Authors:** Thank you for the correction. We will separate againstonchocerciasis to against onchocerciasis.

Patients identified with TT (through surveys, routine consultations in eye care centers, mobile eye screening and management campaigns, during advanced strategies used by some eye care centers, as well as during door-to-door visits during the integrated mass treatment campaigns **against onchocerciasis**, schistosomiasis and geo-helminthiasis in 2015 and 2017) and for whom TT surgery was necessary, were managed.

Line 230 – typo advance strategies

**Authors:** Thank you for the correction. This correction will be incorporated in the revised version.

Patients with TT who present to health centers where eye care services are available are operated on in fixed posts and those screened in **advanced strategies** are managed in the health facility closest to the patient.

Line 332. Please check. I think Togo came before Malawi

**Authors:** Thank you for the correction.

We checked. Indeed, Togo was certified (in May 2022) before Malawi (in September 2022).

These corrections will be integrated in the revised version.

**Reviewer #3:**

Thank you for the opportunity to review a revised version of the article “The elimination of trachoma as a public health problem in Togo: Successes and Challenges”. This is a marked improvement on the previous manuscript and broadly addresses my concerns and comments on the previous version. The title better matches the content of the manuscript. The write-up clearly distinguishes the data submitted to the WHO for certification of trachoma elimination from post-elimination surveillance data collected from the surveillance systems that are in place in a logical and chronological order making the paper very easy to follow and make sense of. The flow of the write-up is now commendable but needs slight editing.

The objective of this review paper is clear with great lessons for other programmes regarding the successes and challenges of the process. The introduction contains adequate background information on the global trachoma elimination programme and the strides made on the African continent.

The programme’s benefit from the broader government policies, strategies, processes, and resources is commendable and well documented. It presents great lessons for countries to learn from in presenting their programmes to governments for support.

At this point, it is clear that the paper is very useful and relevant to the current strategies of the WHO NTD 2030 Roadmap and global trachoma elimination. The lessons presented will be of immense assistance to other programmes, especially in sub-Saharan Africa.

I recommend that paper for publication after the required editorial corrections.

**Authors:** Thank you for this comment. We thank you for your acceptance to review this manuscript.
